# Neutralizing activity against bovine H5N1 HPAIV (clade 2.3.4.4b) in human plasma after seasonal influenza vaccination

**DOI:** 10.1080/22221751.2025.2528539

**Published:** 2025-07-01

**Authors:** Lu Zhang, Georg M. N. Behrens, Amy Kempf, Inga Nehlmeier, Sabine Gärtner, Anna-Sophie Moldenhauer, Luise Graichen, Christine Happle, Michael Winkler, Alexandra Dopfer-Jablonka, Stefan Pöhlmann, Markus Hoffmann

**Affiliations:** a Infection Biology Unit, German Primate Center – Leibniz Institute for Primate Research, Göttingen, Germany; b Faculty of Biology and Psychology, Georg-August-University Göttingen, Göttingen, Germany; c Department of Rheumatology and Immunology, Hannover Medical School, Hannover, Germany; d German Center for Infection Research (DZIF), partner site Hannover-Braunschweig, Hannover, Germany; e CiiM - Centre for Individualized Infection Medicine, Hannover, Germany; f Department of Paediatric Pneumology, Allergy and Neonatology, Hannover Medical School, Hannover, Germany

**Keywords:** H5N1, clade 2.3.4.4b, bovine, neutralization, seasonal influenza vaccination

## Abstract

In 2024, a clade 2.3.4.4b H5N1 highly pathogenic avian influenza virus (HPAIV) emerged in dairy cattle in the United States and spread rapidly to over 1,000 herds across multiple states. At least 41 human infections have occurred through contact with infected cattle, though no fatalities have been reported so far. This raises questions about whether the human innate immune system provides a barrier to bovine H5N1 HPAIV and whether seasonal influenza vaccines offer cross-protection. To address these questions, we used pseudoviruses bearing hemagglutinin (HA) and neuraminidase (NA) from seasonal influenza A or various H5Ny HPAIV strains (from cattle, duck, and seal). Pseudoviruses bearing H5N1 HPAIV HA and NA entered a wide range of mammalian and avian cell lines, including multiple cell lines from the human respiratory tract, while entry into A549 human lung cells was reduced when IFITM proteins were expressed. Additionally, preincubation of pseudovirus particles bearing H5N1 HPAIV HA and NA with plasma from individuals vaccinated with seasonal influenza vaccines inhibited viral entry. Collectively, these results suggest that the human innate immune system imposes a barrier against bovine H5N1 HPAIV infection and that seasonal influenza vaccines can induce cross-neutralizing activity against bovine H5N1 HPAIV.

H5Ny highly pathogenic avian influenza viruses (HPAIV) cause significant mortality in birds [1] and have led to fatal cases in humans and other mammals, though sustained transmission in mammals is rare, except in seals and sea lions [2,3]. This changed in March 2024, when clade 2.3.4.4b H5N1 HPAIV was detected in dairy cattle in the United States, and the virus spread to other animals and humans [4–6]. As of May 09, 2025, 1053 dairy herds across 17 states have been affected (https://www.cdc.gov/bird-flu/situation-summary/mammals.html) and 41/70 human cases of bird flu were linked to exposure to dairy cattle since 2024 (https://www.cdc.gov/bird-flu/situation-summary/index.html). While no fatal human infections from clade 2.3.4.4b bovine H5N1 HPAIV have occurred, concerns remain that the virus could mutate, increasing its infectivity and pathogenicity in humans.

Here, we studied host cell entry of bovine H5N1 HPAIV and investigated whether seasonal influenza virus vaccines can induce cross-neutralizing antibodies. In the absence of a virus isolate, we employed pseudoviruses bearing influenza A virus (IAV) hemagglutinin (HA) and neuraminidase (NA) proteins, since pseudoviruses are a frequently used surrogate system for the analysis of IAV neutralization [7–9]. Further, neutralization titres obtained from pseudovirus particles bearing IAV HA and NA have been demonstrated to correlate well with neutralization titres obtained by hemagglutination inhibition and microneutralization assays carried out with authentic virus [10–12].

For pseudotyping, we utilized the hemagglutinin (HA) and neuraminidase (NA) proteins of the seasonal human IAVs H1N1 A/New York City/PV101028/2024 (clade 6B.1A.5a.2a.1, humanH1N1pp) and H3N2 A/New York City/PV101188/2024 (clade 3C.2a1b.2a.2a.3a.1, humanH3N2pp), and three HPAIV (H5N1 A/duck/Hubei/ZYSYF25/2016, clade 2.3.2.1c [duckH5N1pp], H5N1 A/dairy cattle/Texas/24-008749-001-original/2024, clade 2.3.4.4b [bovineH5N1pp], H5N8 A/seal/Germany-SH/AI05379/2021, clade 2.3.4.4b [sealH5N8pp]).

We first examined the cell line tropism of bovine H5N1 HPAIV using seven human and eight animal cell lines. Control particles bearing vesicular stomatitis virus glycoprotein entered all cell lines with high efficiency (Figure 1(A)). Further, HPAIV H5Ny pseudoviruses entered all cell lines without trypsin treatment while human IAVs required trypsin for HA cleavage (Figure S1) and entry (Figure 1(A)). Surprisingly, humanH1N1pp displayed low entry capacity, which likely stemmed from inefficient particle incorporation of the corresponding HA and NA proteins (Figure S1). Finally, entry of bovineH5N1pp was generally less efficient compared to entry of duckH5N1pp and sealH5N8pp (Figure 1(A)).

**Figure 1.**
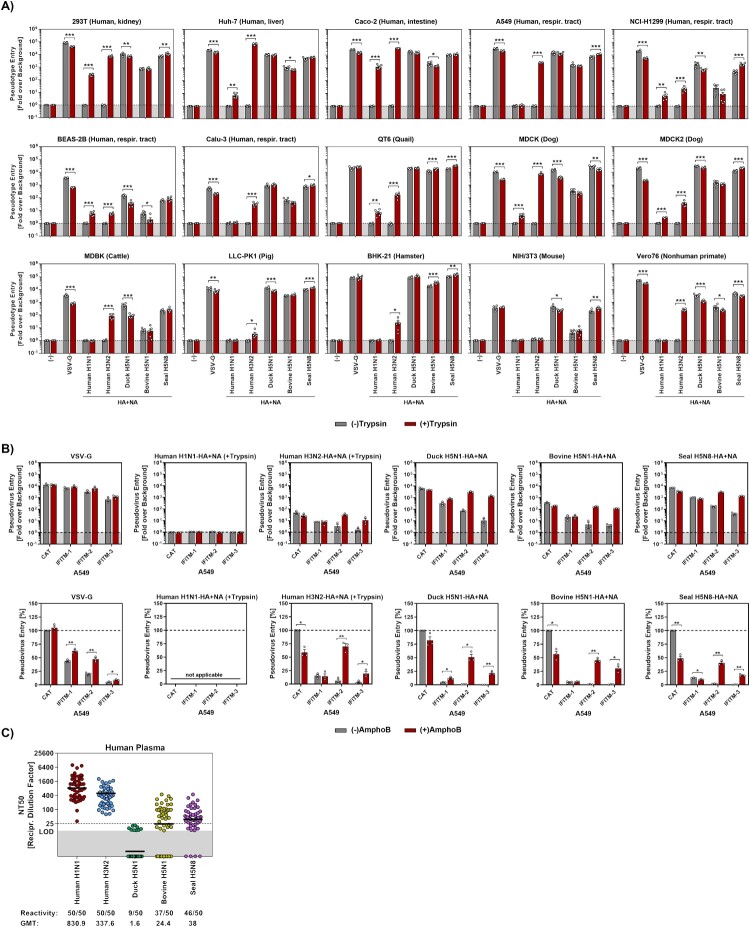
Cell line tropism, IFITM inhibition and neutralization sensitivity of particles bearing clade 2.3.4.4b bovine H5N1 HPAIV HA and NA. (A) Pseudoviruses were trypsin-treated or mock-treated, and inoculated onto the indicated cell lines. Cell entry was analyzed by quantification of luciferase activity in cell lysates. Data represent the mean of six experiments (four technical replicates), and entry was normalized against the background (signals obtained from particles without viral glycoprotein, set as 1). Error bars represent the standard error of the mean (SEM). (B) A549 cells stably expressing IFITM1, IFITM2, IFITM3, or CAT (chloramphenicol acetyltransferase) were pre-treated with amphotericin B (AmphoB, 2.5 µM) or mock-treated, before being inoculated with pseudoviruses. Data represent the mean of three experiments (four technical replicates), and cell entry was either normalized against the background (upper panels) or against signals obtained for A549-CAT cells (set as 100%, lower panel). Error bars represent the SEM. (C) Pseudoviruses were preincubated with human plasma (*n* = 50) and inoculated onto Caco-2 cells. Entry was normalized against entry in the absence of plasma (0% inhibition) and the neutralizing titre 50 values were determined. Data represent individual NT50 values and geometric mean titres (GMT) from a single experiment (four technical replicates). Information below the graphs indicate reactivity rates and GMT values. For graphical reasons samples yielding an NT50 value below the limit of detection (LOD, 12.5) were assigned a value of 1. The dashed line indicates the lowest plasma dilution tested. For panels A and B, statistical significance was analyzed by two-tailed Student's *t*-test with Welch correction (*, *p* ≤ 0.05; **, *p* ≤ 0.01; ***, *p* ≤ 0.001).

The interferon (IFN) system plays a key role in antiviral defence. Proteins encoded by interferon-stimulated genes exert antiviral activities [13], including interference with influenza A virus cell entry by interferon-induced transmembrane (IFITM) proteins [14]. We tested whether IFITM proteins block cell entry of bovineH5N1pp using A549 cells stably expressing IFITM1, IFITM2, IFITM3, or CAT (chloramphenicol acetyltransferase, control). IFITM-expression inhibited cell entry of all pseudotypes tested with IFITM3 exerting the most potent inhibitory activity. Amphotericin B can rescue entry from inhibition by IFITM proteins [15] and indeed augmented cell entry of humanH3N2pp, duckH5N1pp, bovineH5N1pp and sealH5N8pp into IFITM-2- or -3 expressing cells (Figure 1(B)). In contrast, entry of particles bearing IAV-HA+NA – but not VSV-G – into CAT-expressing control cells was reduced rather than enhanced (Figure 1(B)), indicating that under the experimental conditions used, amphotericin B can exert anti-IAV activity independent of IFITM-mediated restriction for at present unclear reasons.

Finally, we examined whether antibodies induced from seasonal influenza vaccines cross-neutralize bovine H5N1 HPAIV. A pseudovirus neutralization assay with human plasma samples obtained post-vaccination revealed robust neutralization of humanH1N1pp (GMT = 830.9) and humanH3N2pp (GMT = 337.6). BovineH5N1pp (GMT = 24.4) and sealH5N8pp (GMT = 38) were moderately neutralized, while duckH5N1pp showed low neutralization (GMT = 1.6; Figure 1(C) and Figure S2). Depletion of immunoglobulin G antibodies reduced neutralization, confirming that antibodies were responsible for the inhibition of cell entry (Figure S3).

## Discussion

We report that bovine H5N1 HPAIV exhibits a cell line tropism similar to other H5Ny HPAIV and is inhibited by IFITM proteins. Moreover, we provide evidence that seasonal influenza vaccines induce cross-neutralizing antibodies against bovine H5N1 HPAIV. While our results await confirmation with authentic virus, our findings align with a study by Gioia *et al.*, demonstrating that seasonal influenza vaccines induce neutralizing antibodies against clade 3 H5N1 HPAIV [16]. Additionally, Sanz-Muñoz et al. obtained evidence for the existence of low level basal heterosubtypic immunity against H5N1 HPAIV subtypes in humans that could be increased by seasonal influenza vaccination using a recombinant H1N1 influenza virus expressing HA and NA proteins of H5N1 HPAIV A/chicken/Egypt/F71-F114C/2022 (clade 2.3.4.4b) [17] – however, bovine H5N1 HPAIV was not tested. Collectively, our results suggest that the innate immune system imposes a barrier against bovine H5N1 HPAIV infection and that seasonal influenza vaccines induce cross-neutralizing activity against bovine H5N1 HPAIV. Whether this cross-neutralization can protect humans against infection by bovine H5N1 HPAIV and/or severe disease development requires further research.

## Supplementary Material

SI_Zhang et al_Bovine H5N1_R2_clean.docx

## Data Availability

The authors confirm that the data supporting the findings of this study are available within the article and/or its supplementary materials.
