# Supplementary material for: Neutralizing activity against bovine H5N1 HPAIV (clade 2.3.4.4b) in human plasma after seasonal influenza vaccination
Source: Emerg Microbes Infect. 2025 Jul 1;14(1):2528539. doi: 10.1080/22221751.2025.2528539 (PMC12302390; doi:10.1080/22221751.2025.2528539)
Supplement: SI_Zhang et al_Bovine H5N1_R2_clean.docx [file TEMI_A_2528539_SM0090.docx]

# Supplementary methods

**Cell culture and transfection**

All cell lines were cultivated at a temperature of 37 °C in a humidified atmosphere containing 5% CO_2_. 293T (human kidney, female; DSMZ catalogue no. ACC-635, RRID:CVCL 0063), Huh-7 (human liver, male; JCRB Cat# JCRB0403; RRID:CVCL 0336; kindly provided by Thomas Pietschmann), MDCK (dog kidney, female; ATCC catalogue no. CCL-34, RRID:CVCL_0422), MDCK2 (dog kidney, female; ATCC catalogue no. CRL-2936, RRID:CVCL_B034; kindly provided by Georg Herrler), MDBK (cattle kidney, male, ATCC catalogue no. CCL-22, RRID:CVCL_0421; kindly provided by Georg Herrler), LLC-PK1 (pig kidney, male; ATCC catalogue no. CL-101, RRID:CVCL_0391; kindly provided by Georg Herrler), BHK-21 (Syrian golden hamster kidney, male; ATCC catalogue no. CCL-10, RRID:CVCL_1915; kindly provided by Georg Herrler), NIH/3T3 (mouse embryo, male; ATCC catalogue no. CRL-1658, RRID:CVCL_0594), and Vero 76 cells (African green monkey kidney, female; ATCC catalogue no. CRL-1586; RRID:CVCL 0574; kindly provided by Andrea Maisner) were cultivated in Dulbecco's modified Eagle medium (PAN-Biotech), supplemented with 10% fetal bovine serum (FBS, Biochrom), and the antibiotics penicillin and streptomycin at final concentrations of 100 U/ml and 0.1 mg/ml, respectively (pen/strep, PAN-Biotech). Caco-2 cells (human intestine, male; ATCC catalogue no. HTB-37, RRID:CVCL_0025; kindly provided by Hassan Y. Naim) were cultured in minimum essential medium (Thermo Fisher Scientific) supplemented with 10% FBS, pen/strep, 1% non-essential amino acid solution (PAA) and 1 mM sodium pyruvate (PAN-Biotech). Calu-3 (human lung, male; ATCC catalogue no. HTB-55, RRID:CVCL_0609; kindly provided by Stephan Ludwig) were cultured in DMEM/F-12 medium (Thermo Fisher Scientific) supplemented with 10% FBS, pen/strep, 1% non-essential amino acid solution and 1 mM sodium pyruvate. A549 cells (human lung, male; CRM-CCL-185, ATCC, RRID:CVCL_0023; kindly provided by Georg Herrler) were cultured in F-12 medium (Thermo Fisher Scientific) supplemented with 10% FBS, pen/strep, 1% non-essential amino acid solution and 1 mM sodium pyruvate. NCI-H1299 (human lung, male; ATCC catalogue no. CRL-5803, RRID:CVCL_0060; kindly provided by Christian Drosten) and BEAS-2B cells (human lung/bronchus, male; ATCC catalogue no. CRL-3588, RRID:CVCL_0168) were cultured in Roswell Park Memorial Institute (RPMI) medium (PAN-Biotech) supplemented with 10% FBS, pen/strep, 1% non-essential amino acid solution and 1 mM sodium pyruvate. QT6 (quail fibrosarcoma, unspecified sex; ATCC catalogue no. CRL-1708, RRID:CVCL_3451) were cultivated in Ham's F-12K medium (Thermo Fisher Scientific) supplemented with 10% tryptose phosphate broth (Thermo Fisher Scientific), 5% FBS and pen/strep. A549 cells stably expressing IFITM1, IFITM2, IFITM3, or CAT (chloramphenicol acetyltransferase) were generated by retroviral transduction and selection with puromycin. The cell lines used in this study were validated by STR analysis, amplification and sequencing of a cytochrome c oxidase gene fragment, microscopic examination, and/or their specific growth characteristics. Moreover, all cell lines were regularly screened for mycoplasma contamination. The calcium phosphate precipitation method was used for transfection of 293T cells.

**Expression plasmids**

Expression plasmids for codon-optimized (for codon-usage in human cells) hemagglutinin (HA) and neuraminidase (NA) proteins of H1N1 A/New York City/PV101028/2024 (clade 6B.1A.5a.2a.1, GISAID ID: EPI_ISL_19091263; shared by CEIRS Data Processing and Coordinating Center, Center for Research on Influenza Pathogenesis), H3N2 A/New York City/PV101188/2024 (clade 3C.2a1b.2a.2a.3a.1, GISAID ID: EPI_ISL_19091264; shared by CEIRS Data Processing and Coordinating Center, Center for Research on Influenza Pathogenesis), HPAIV H5N1 A/duck/Hubei/ZYSYF25/2016 (clade 2.3.2.1c, GenBank: KY415618.1 and KY415706.1), HPAIV H5N1 A/dairy cattle/Texas/24-008749-001-original/2024 (clade 2.3.4.4b, GISAID ID: EPI_ISL_19014384; shared by National Veterinary Services Laboratories - USDA), and HPAIV H5N8 A/seal/Germany-SH/AI05379/2021 (clade 2.3.4.4b, GISAID ID: EPI_ISL_4805936; shared by Tierärztliche Hochschule Hannover and Friedrich-Loeffler-Institut) were generated by Gibson assembly (An alignment of the HA protein sequences can be found in **Figure S4**). For each gene, two overlapping DNA strings (commercially purchased, Thermo Fisher Scientific, sequences available upon request) were mixed with linearized (BamHI/XbaI-digested) pCG1 plasmid (a kind gift of Roberto Cattaneo, Mayo Clinic College of Medicine, Rochester, MN, USA) and GeneArt™ Gibson Assembly HiFi Master Mix (Thermo Fisher Scientific), and samples were incubated for 45 min at 50 °C, before the mixtures were transformed into one-shot OmniMAX 2 T1 competent *Escherichia coli* bacteria (Thermo Fisher Scientific) and plated onto LB agar plates containing 50 µg/ml of ampicillin. The following day, bacterial colonies were screened for presence of the insert by PCR, and positive clones were expanded for plasmid preparation. In addition, expression plasmids coding for HA proteins harboring a C-terminal V5-epitope tag (sequence: GKPIPNPLLGLDST) as well as NA proteins harboring a C-terminal FLAG-epitope tag (sequence: DYKDDDDK) were generated by PCR using a reverse primers containing the respective epitope-coding sequence. Finally, the integrity of all sequences was confirmed by Sanger sequencing (Microsynth SeqLab). Further information on the HA and NA protein sequences can be obtained from the GISAID (Global Initiative on Sharing All Influenza Data) EpiFlu^TM^ database (<https://gisaid.org/>). The expression plasmid pCAGGS-VSV-G has been described before[1].

**Pseudovirus particle production and cell entry**

A pseudovirus particle system based on a replication-restricted vesicular stomatitis virus was employed to study host cell entry of influenza A viruses and its neutralization. For this, 293T cells were transfected to express the respective HA and NA proteins, VSV-G (positive control) or empty pCG1 plasmid (negative control). At 24 h posttransfection, cells were inoculated with VSV-G-transcomplemented VSV*ΔG(FLuc) (kindly provided by Gert Zimmer)[2] at a multiplicity of infection of 3 for 1 h, before the inoculum was aspirated and cells were washed with phosphate-buffered saline (PBS). Thereafter, cells transfected with HA and NA expression vectors or empty plasmid received fresh culture medium supplemented with anti-VSV-G antibody (culture supernatant from I1-hybridoma cells; ATCC no. CRL-2700), while cells transfected with VSV-G expression vector received medium without antibody. Following an incubation period of 16-18 h, the pseudovirus-containing cell culture supernatants were collected, clarified from debris by centrifugation (4,000 x g, 10 min) and the clarified supernatants were split into two separate tubes. Next, bovine trypsin (Sigma-Aldrich, 10 µg/ml final concentration) was added to one of the two tubes of each pseudovirus preparation and the samples were incubated for 30 min at 37 °C, before soybean trypsin inhibitor (Sigma-Aldrich, 20 µg/ml final concentration) was added. Finally, all samples were aliquoted and stored at -80 °C until further use.

In order to study cell tropism of HA+NA protein-bearing pseudovirus particles, target cells were seeded into 96-well plates. On the next day, identical volumes of pseudovirus particles were added onto target cells and incubated for 16-18 h, before the culture medium was aspirated and PBS containing 0.5% Tergitol™ 15-S-9 (Carl Roth) was added to induce cell lysis. After an incubation phase of 30 min, cell lysates were transferred into white 96-well plates and luminescence was recorded using a Hidex Sense plate luminometer (Hidex) following addition of luciferase substrate (Beetle-Juice, PJK).

**Sodium dodecyl-sulfate polyacrylamide gel electrophoresis (SDS-PAGE) and immunoblot**

Pseudovirus particles bearing HA proteins containing a C-terminal V5-epitope tag (HA-V5) as well as NA proteins containing a C-terminal FLAG-epitope tag (NA-FLAG) were incubated in the presence or absence of trypsin (20 µg/ml for 30 min at 37 °C), before being loaded on a sucrose cushion (20% w/v, 50 µl) and centrifuged at 21,000 rpm for 90 min at 4 °C. Following centrifugation, the supernatant was removed except for the sucrose cushion, which was mixed with SDS sample buffer (0.03 M Tris-HCl, 10% glycerol, 2% SDS, 5% beta-mercaptoethanol, 0.2% bromophenol blue, 1 mM EDTA) and heated to 96 °C for 10 min. Next, samples were loaded on polyacrylamide gels, subjected to SDS-PAGE and afterwards transferred onto nitrocellulose membranes (Hartenstein). Next, the membranes were blocked with blocking buffer (PBS with 0.05% Tween-20 and 5 g/l skim milk powder) for 1 h at room temperature and subsequently incubated overnight at 4 °C with primary antibody diluted in PBS/0.05% Tween-20. Membranes were probed with anti-V5 (V5-tag Polyclonal antibody, mouse, 1:1000, Proteintech, Cat: 14440-1-AP) or anti-FLAG (Monoclonal ANTI-FLAG® M2, mouse, 1:1000, Sigma-Aldrich, Cat: F1804) antibodies for detection of HA-V5 and NA-FLAG, respectively. In addition, VSV-M (vesicular stomatitis virus matrix protein, loading control) was detected using an anti-VSV-M antibody (anti-VSV-M [23H12] antibody, mouse, 1:1000, Kerafast, Cat: EB0011). Following incubation with primary antibody, membranes were washed three times with PBS/0.05% Tween-20 for 15 min at room temperature and subsequently incubated with PBS/0.05% Tween-20 containing the secondary antibody, goat IgG anti-mouse IgG (H+L)-HRPO (1:2000, Dianova, Cat: 115-035-045), for 1 h at room temperature. Finally, membranes were washed three times with PBS/0.05% Tween-20 for 15 min at room temperature, before being developed using a chemiluminescence substrate (Westar Antares, Cyanagen). Imaging was performed using the Azure600 imaging system and the AzureSpot Pro software.

**Amphotericin B-treatment of target cells**

In order to alleviate the antiviral activity of IFITM proteins, A549 cells stably expressing IFITM1, IFITM2, IFITM3, or CAT were incubated for 2 h in the presence of 2.5 µM amphotericin B (Sigma-Aldrich) diluted in culture medium, before pseudovirus particles were added.

**Neutralization assay**

Neutralization assays were conducted based on a modified protocol that was originally developed for neutralization assays involving pseudovirus particles bearing SARS-CoV-2 spike proteins[3]. In brief, Caco-2 cells were seeded into 96-well plates and allowed to reach confluency. Next, pseudovirus particles were pre-incubated (30 min at 37 °C) with four-fold serial dilutions of heat-inactivated (56 °C, 30 min) human plasma (starting dilution = 1:25), before the mixtures were added to the cells. At 16-18 h postinoculation, luminescence was measured as described in the previous paragraph. The neutralization efficiency was calculated based on the relative inhibition of pseudovirus entry, with entry of pseudovirus particles incubated in the absence of serum/plasma set as 0% inhibition. Further, a non-linear regression model was used to calculate the serum/plasma dilutions causing half-maximal inhibition (neutralizing titer 50, NT50). Of note, serum/plasma samples that yielded an NT50 value lower than 12.5 were considered negative and were assigned an NT50 value of 1.

**Human plasma samples**

Human plasma samples (n = 50; male to female ration: 17:33, age range: 23-68 years [median = 52 years]; see also **Figure S5**) were obtained at a median of three months after influenza vaccination in the winter season of 2023/24 and sampling was performed as part of the of the COVID-19 Contact (CoCo) Study (German Clinical Trial Registry, DRKS00021152). For that season, Vaxigrip Tetra® (Sanofi Winthrop Industry, France) was vaccinated at Hannover Medical School, people >60 years of age (20% of our cohort) received the tetravalent vaccine Efluelda® 2023/24 (Sanofi Pasteur Inc. USA). Both vaccines contain the following strain composition: Influenza A/Victoria/4897/2022 (H1N1)pdm09 pandemic-like strain, Influenza A/Darwin/9/2021 (H3N2) pandemic-like strain, Influenza B/Austria/1359417/2021 pandemic-like strain, Influenza B/Phuket/3073/2013 pandemic-like strain. The collection and analysis of human plasma samples was conducted following approval granted by the research ethics committee of the Institutional Review Board of Hannover Medical School (8973_BO_K_2020). All participants provided written informed consent prior to the use of plasma samples for research and received no compensation.

**Immunoglobulin G depletion**

Selected plasma samples with neutralizing activity against bovineH5N1_pp_ (n = 9) were split into two tubes. One sample was left untreated (untreated sample) while the other sample was mixed with 50 µl protein A-sepharose (Sigma-Aldrich, 50% v/v in PBS) and incubated overnight at 4 °C on a rotating disc. Thereafter, the protein A-sepharose was pelleted by centrifugation (2,500 x g, 10 min, 4 °C) and the supernatant was transferred into a fresh tube (IgG-depleted sample). Next, untreated and IgG-depleted samples were subjected to neutralizations assays.

**Data analysis**

Data analysis was performed with the help of Microsoft Excel (part of Microsoft Office Professional Plus, version 2016, Microsoft Corporation) and GraphPad Prism version 8.3.0 (GraphPad Software). Statistical significance was assessed by two-tailed Student’s t-test with Welch correction or Wilcoxon matched-pairs signed rank test and only p values of 0.05 or lower were considered statistically significant (not significant, p > 0.05; *, p ≤ 0.05; **, p ≤ 0.01; ***, p ≤ 0.001).

# Limitations of the study

The following limitations apply to our study. First, we utilized cell lines (including only a single cell line of bovine origin) and pseudovirus particles bearing influenza A virus HA and NA protein to study influenza A virus host cell entry and neutralization, which is why our data await confirmation with authentic influenza A virus and primary cell cultures. Second, the use of pseudoviruses does not allow assessment of viral replication in target cells and the impact of neutralizing antibodies on multicycle replication since this system is limited to a single round of infection. Third, while our results suggest that human IFITM proteins can restrict bovine H5N1 HPAIV cell entry into A549 cells, we did not test whether this is also true for additional human cell lines or bovine cell lines expressing bovine IFITM proteins. Fourth, since our study aims to provide a rapid assessment on the ability of seasonal influenza vaccines to induce antibodies that cross-neutralize bovine H5N1 HPAIV (clade 2.3.4.4b), the sample size is relatively small and precludes investigation of the impact of biological factors (e.g., age and gender) on neutralization. Fifth, since all human plasma samples were collected within six months of vaccination, we cannot make any statement on bovine H5N1 HPAIV neutralization after extended time periods post vaccination. Sixth, although it is highly unlikely that study participants with neutralizing activity against HPAIV H5Ny may have been infected by such viruses in the past – and thus H5N1- and H5N8-specific neutralizing activity in their plasma may stem from previous infection rather than vaccination – this hypothesis could not be tested.

# Supplementary references

[1] Brinkmann C, Hoffmann M, Lubke A, et al. The glycoprotein of vesicular stomatitis virus promotes release of virus-like particles from tetherin-positive cells. PloS one. 2017;12(12):e0189073.

[2] Berger Rentsch M, Zimmer G. A vesicular stomatitis virus replicon-based bioassay for the rapid and sensitive determination of multi-species type I interferon. PLoS One. 2011;6(10):e25858.

[3] Arora P, Zhang L, Kruger N, et al. SARS-CoV-2 Omicron sublineages show comparable cell entry but differential neutralization by therapeutic antibodies. Cell host & microbe. 2022;30(8):1103-1111 e6.

# Supplementary figures

**
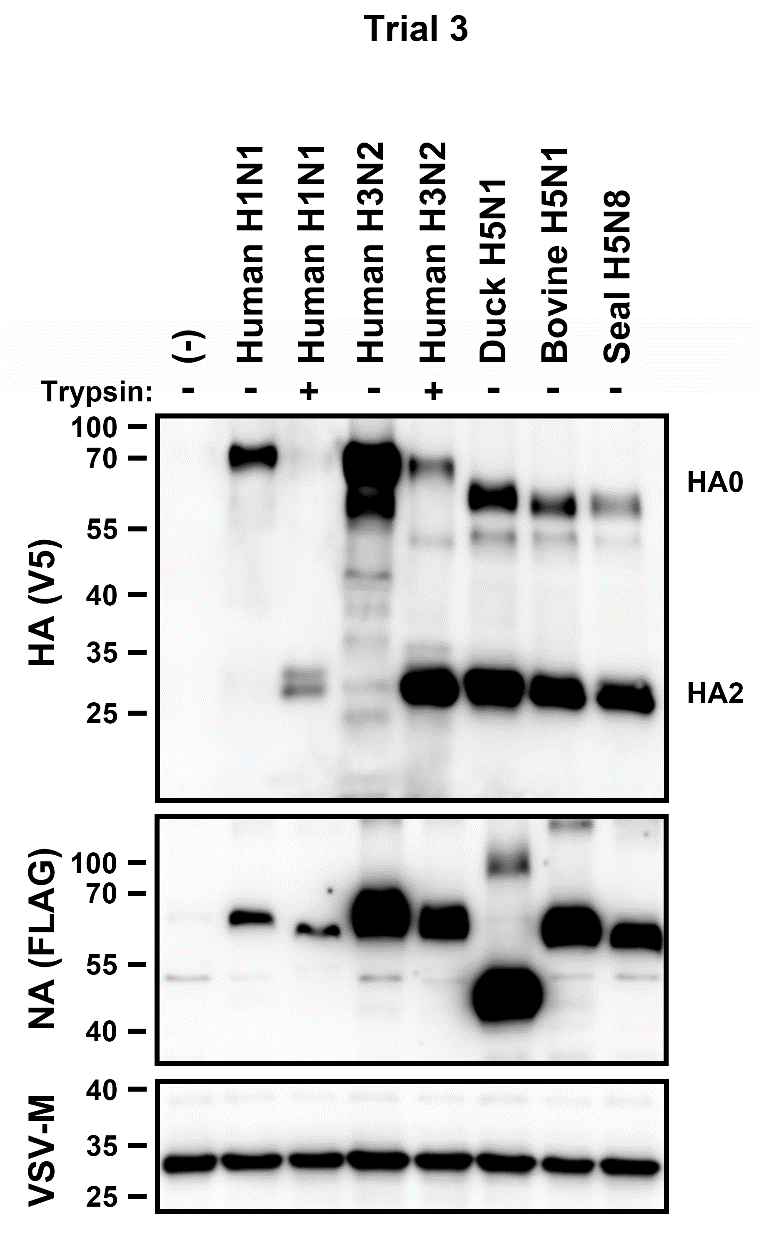
**

**Figure S1: HA and NA particle incorporation and proteolytic HA cleavage.**

Pseudovirus particles bearing V5-tagged HA (HA-V5) and FLAG-tagged NA (NA-FLAG) proteins of the indicated influenza A viruses were incubated in the presence or absence of trypsin (20 µg/ml for 30 min at 37 °C) and subsequently subjected to SDS-PAGE and immunoblot. Particles bearing neither HA nor NA proteins served as control. Detection of HA-V5, NA-FLAG and vesicular stomatitis virus matrix protein (VSV-M, loading control) was carried out with primary antibodies directed against the V5-epitope, FLAG-epitope or VSV-M, respectively, and horseradish peroxidase-coupled secondary antibodies. Representative immunoblot data are shown and results were confirmed in a total of three biological replicates. Bands representing uncleaved (HA0) and cleaved (HA2) HA proteins are indicated. Numerical values on the left indicate the molecular weight in kilodalton (kDa).

**
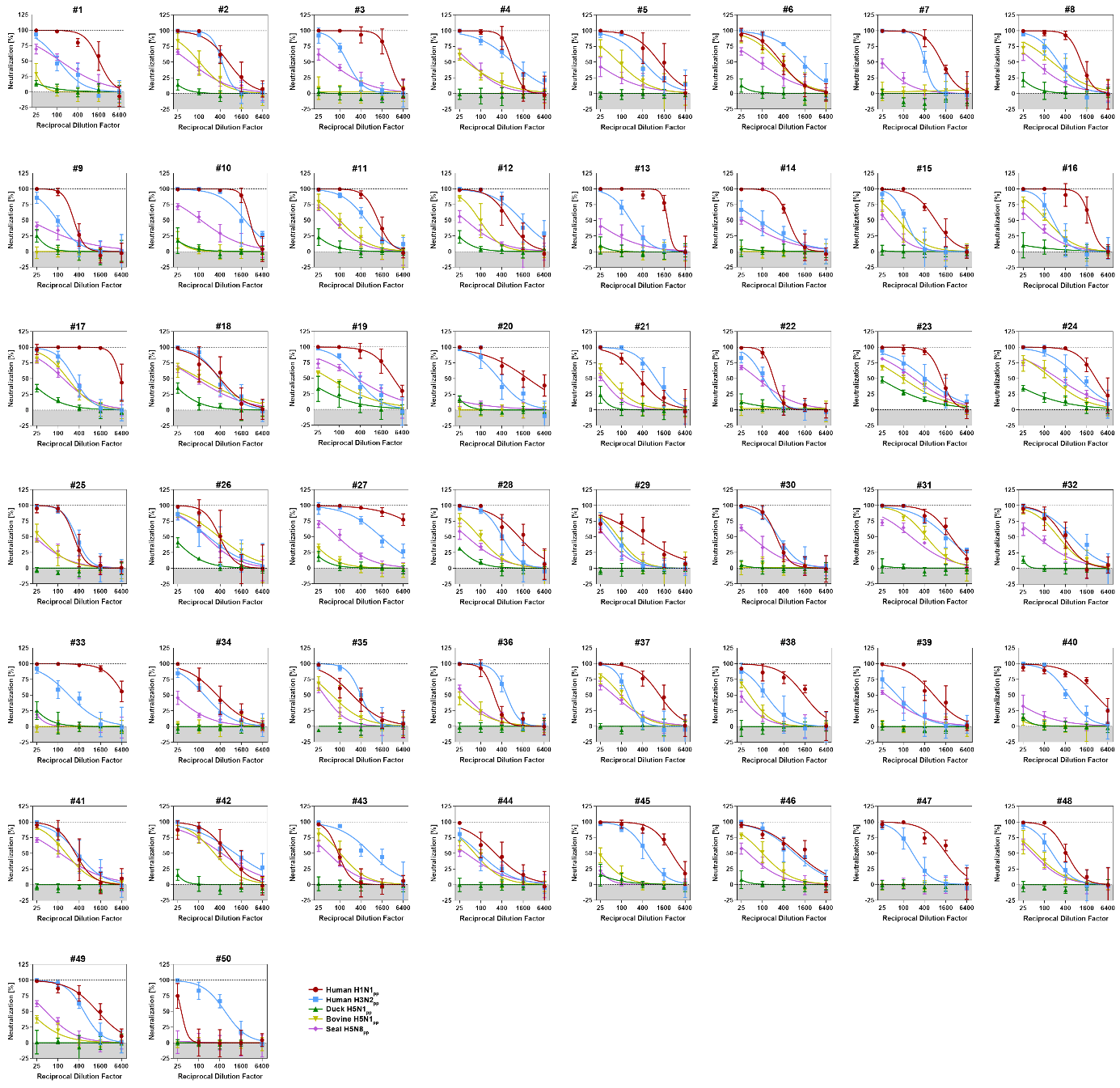
**

**Figure S2: Individual neutralization data for human plasma samples.**

Individual neutralization data for pseudovirus particles bearing the HA and NA proteins of the indicated influenza A viruses that were incubated with human plasma.


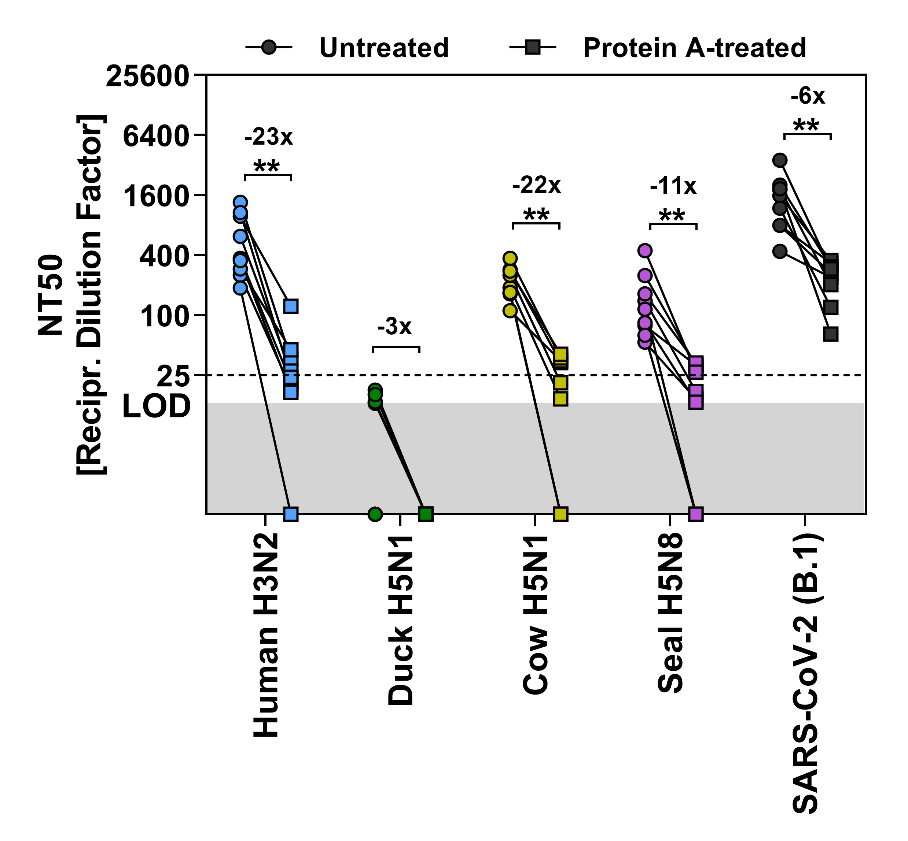


**Figure S3: Immunoglobulin G depletion reduces neutralizing activity in human plasma against bovine H5N1 HPAIV.**

Nine human plasma (n = 9) were divided into two samples of which one was left untreated while the other was incubated overnight at 4 °C in the presence of protein A-sepharose and immunoglobulin G (IgG) bound to protein A sepharose was subsequently removed by centrifugation. Next, untreated and IgG-depleted (protein A-treated) plasma samples were subjected to pseudovirus particle neutralization assays using Caco-2 cells and particles bearing the indicated influenza A virus HA and NA proteins or the SARS-CoV-2 spike protein (B.1 lineage, control). Cell entry was normalized against entry in the absence of serum/plasma (0% inhibition) and the neutralizing titer 50 values were determined. Data represent individual NT50 values from a single experiment (four technical replicates) with lines connecting the corresponding untreated and IgG-depleted (protein A-treated) plasma samples. Numerical values indicate the mean fold reduction in NT50 values following IgG-depletion. Statistical significance was analyzed by Wilcoxon matched-pairs signed rank test (*, p ≤ 0.05; **, p ≤ 0.01; ***, p ≤ 0.001). Of note, for graphical reasons samples yielding an NT50 value below the limit of detection (LOD, 12.5 = 50% of the lowest plasma dilution tested) were assigned a value of 1.


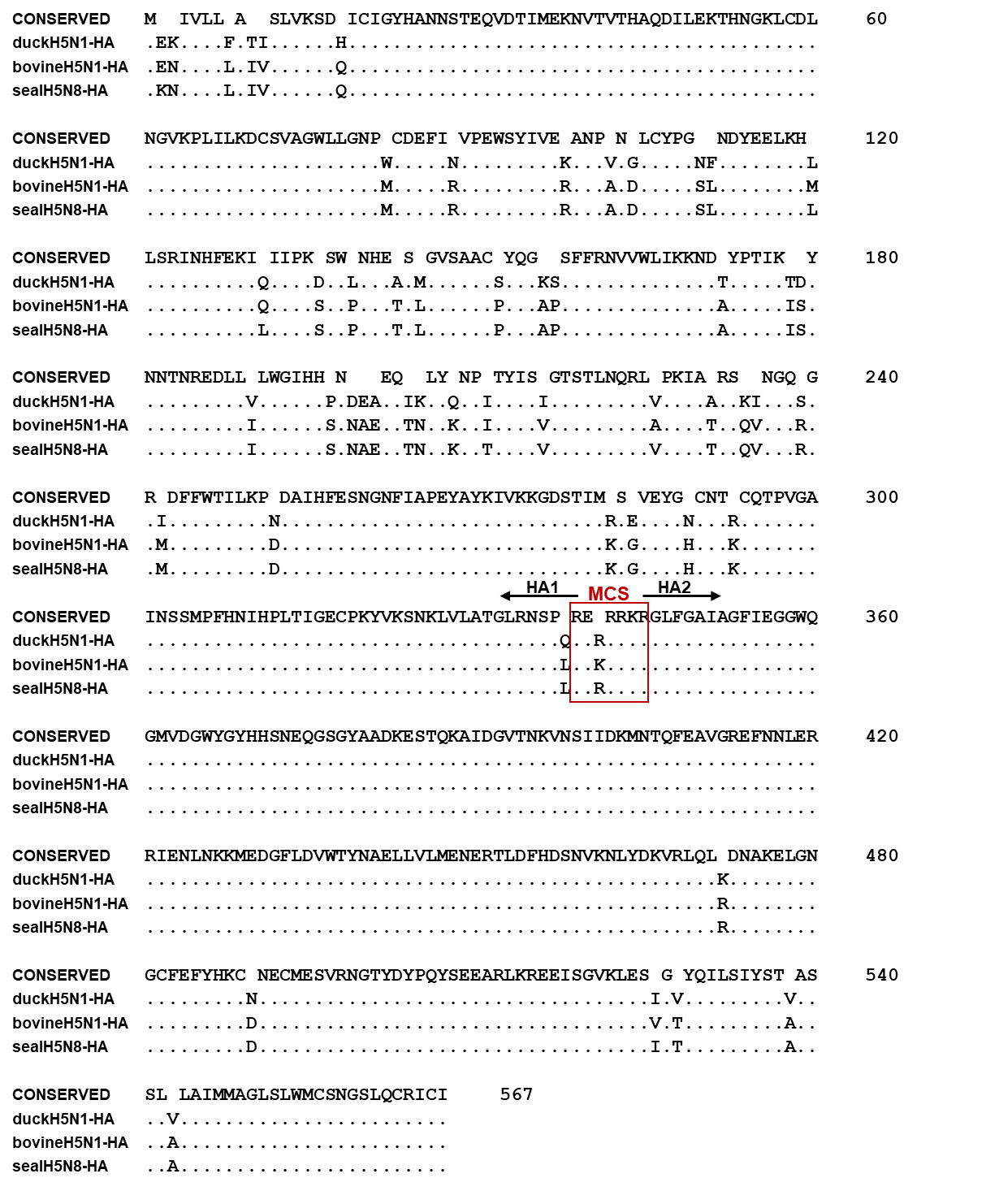


**Figure S4: Amino acid sequence alignment for the HA proteins of duckH5N1, bovineH5N1, and sealH5N8.**

Protein sequences for the indicated HA proteins were aligned using the Clustal Omega tool (<https://www.ebi.ac.uk/jdispatcher/msa/clustalo>). Conserved amino acid residues are indicated. The multibasic cleavage site (MCS) that is located between the HA1 and HA2 subunits is highlighted by a red frame.


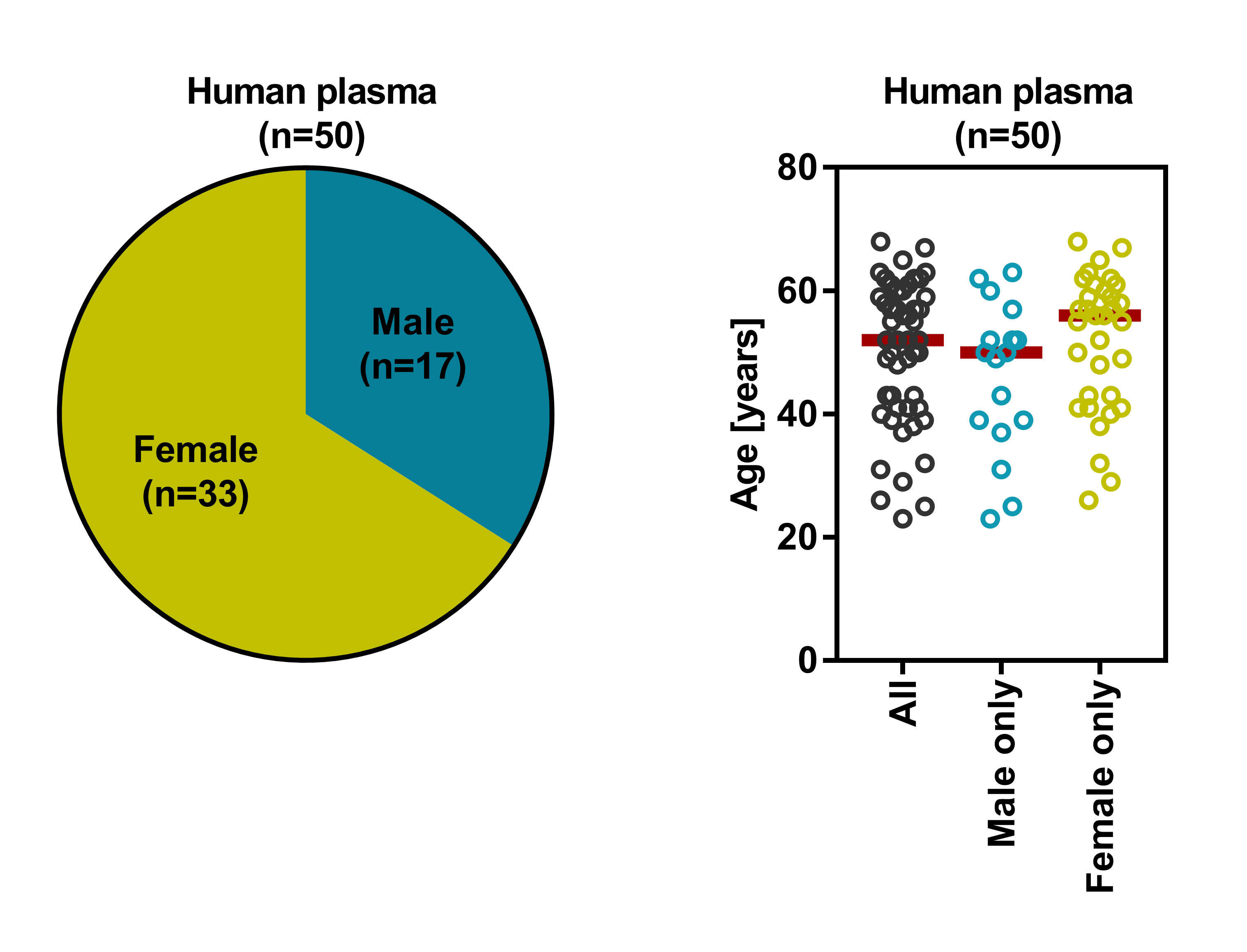


**Figure S5: Information on human plasma donors.**

Information on biological sex (left) and age (right, red lines represent medians) of human plasma donors.
